# Impact of Reactive Balance Training on a Perturbation Treadmill on Physical Performance in Geriatric Patients:Results of a Single-Center, Assessor Blinded Randomized Controlled Trial

**DOI:** 10.3390/jcm13195790

**Published:** 2024-09-28

**Authors:** Alexander Petrovic, Rainer Wirth, Christiane Klimek, Gero Lueg, Diana Daubert, Chantal Giehl, Ulrike Sonja Trampisch

**Affiliations:** Department of Geriatric Medicine, Marien Hospital Herne, Ruhr University Bochum, 44625 Herne, Germany; alexander.petrovic@elisabethgruppe.de (A.P.); rainer.wirth@elisabethgruppe.de (R.W.); christiane.klimek@elisabethgruppe.de (C.K.); gero.lueg@elisabethgruppe.de (G.L.); diana.daubert@elisabethgruppe.de (D.D.); chantal.giehl@elisabethgruppe.de (C.G.)

**Keywords:** accidental falls, fall risk, accident prevention, fall prevention, exercise, training intervention, randomized controlled trial

## Abstract

**Background/Objectives:** Falls and related injuries are a frequent and serious health problem in older persons. Among the various strategies, different forms of active physical training, in particular, have demonstrated success in reducing fall risk. A task-specific training approach is perturbation-based training of reactive balance. Performing this training modality on a perturbation treadmill, secured with a safety harness, is an innovative new approach facilitating task-specific training with unannounced perturbations in a safe environment. The aim of this study was to investigate the feasibility and effectiveness of this specific training in multimorbid older hospitalized patients with prefrailty and frailty. **Methods:** The trial was conducted as a prospective single-center, assessor-blinded randomized controlled trial. A total of 127 acute-care geriatric hospitalized patients were enrolled in a program either involving a minimum of 60 min perturbation-based treadmill training or treadmill training without perturbations on the identical device and for a comparable training period. **Results:** Participants were 81 ± 6 years old (64% female) with a baseline FRAIL Scale, SPPB, and MoCA scores of 3.5 ± 1.6, 8.3 ± 2.6, and 21 ± 5 points, respectively. The training was performed on six occasions with an average total training period of 89 min during a mean hospital stay of 17 ± 3 days. Between the baseline and up to 2 days after the last training, the Short Physical Performance Battery score, which was considered the primary endpoint, improved by 1.4 ± 2.1 points in the intervention group compared to 0.5 ± 1.7 in the control group, with a 0.9-point difference between the groups (*p* < 0.001). **Conclusions**: a relatively short training period of approximately 90 min on a perturbation treadmill led to a significant and clinically meaningful increase in the physical performance of frail and prefrail hospitalized geriatric patients. However, its effectiveness in reducing fall risk is yet to be proven in this population.

## 1. Introduction

Falls and fall-related injuries are very common in older persons and represent a major and increasing health issue. One in three persons over the age of 65 experiences one or more falls per year [1,2,3]. Among those above the age of 80 years, 50% experience a fall at least once per year [4]. Fall-associated injuries, such as fractures, and fear of falling generate severe consequences for the affected persons. Consequently, many strategies have been applied to lower fall risk and avoid associated injuries in the older population [5]. Different training modalities have been evaluated, highlighting the perturbation-based training of reactive balance, which has gained popularity in recent years [6]. By means of unannounced external stimuli, in the form of external perturbations, participants are forced to perform reactive compensatory movements to recover balance. The training appears to be highly effective under laboratory conditions, considering the relatively low training volume [7,8]. In the initial trial by Pai and colleagues, a single training session with 24 unannounced slip perturbations was capable of reducing the fall rate in the future year by 50% [9]. Different approaches for applying adequate perturbations have been implemented. During perturbation training on a treadmill, it is possible to apply frequent slip and trip perturbations while walking; this allows for the individualized task-specific training of the sensorimotor system to cope with external gait perturbations. The special feature of balance training on a perturbation treadmill mainly lies in the possibility of quick changes in the direction and intensity of unannounced perturbations, which are adapted to the individual limits of balance control [6]. A fall reduction of up to 46% after successful perturbation training was described in a 2015 meta-analysis [10]. This accounts for a doubling of the effect compared to conventional fall training programs [11], while the time spent with training is minimized substantially. Another meta-analysis from 2021 by Kim et al. considered perturbation training in general to be a highly effective form of fall prevention training currently available [12].

The current body of research shows clear evidence for the effectiveness of perturbation-based balance training in otherwise healthy older adults [9,13,14]. Respective data on diseased subjects are sparse, and data on multimorbid and frail geriatric patients are missing, although they entail the highest fall risk [15,16]. Currently, up to what level of frailty and cognitive impairment can reactive balance training on a perturbation treadmill be successfully performed, and whether it is effective in terms of improvement in physical performance and a reduction in falls in this population, remain unclear. Therefore, we conducted the present prospective randomized controlled trial in mostly frail and multimorbid older hospitalized patients with fall risk. Here, we report the immediate results on physical performance after the termination of recruitment, as the Short Physical Performance Battery (SPPB) total score is independently associated with falls in older patients and should be used for fall risk assessment [17]. Data on falls will be reported separately after the termination of the 12-month follow-up period. The purpose of this study is to also provide a proof of concept of this training modality in prefrail and frail multimorbid older hospitalized patients.

## 2. Materials and Methods

### 2.1. Design and Setting

This single-center, two-group, assessor-blinded randomized controlled trial (RCT) was conducted in an acute-care geriatric hospital department. Patients admitted to the geriatric acute-care ward as inpatients or day clinic inpatients between October 2019 and December 2022 were recruited. The study was approved by the Ethics Committee (19-6616-BR, approved on 7 June 2019). The trial was registered with German Clinical Trials (DRKS00024637).

### 2.2. Patients’ Recruitment

A total of 138 patients were recruited during hospital stay. Inclusion criteria were patients aged 65 years and older who had experienced at least one fall in the past 365 days, with a body weight below 150 kg, and they had to be able to walk and physically be able to complete the training on the treadmill. Non-inclusion criteria were those with a probable hospital stay in the geriatric department of less than two weeks, total hip replacement within the last 4 weeks, severe pain when walking, paralysis or amputation of a lower limb, and inability to follow instructions or to provide written informed consent.

### 2.3. Procedure

Patients who met the inclusion criteria were informed about the study procedure by a physician, and a baseline assessment was performed by a study assistant. Patients were informed about the follow-up appointments three, six, and twelve months after baseline, which were conducted by telephone. The data of study participants were stored in pseudonymized form.

### 2.4. Randomization and Blinding

Patients meeting the inclusion criteria and willing to participate were reported to the study secretariat. There, randomization (1:1) was generated by an independent employee of the department for each patient individually using an online random number generator (https://www.agitos.de/zufallsgenerator/, accessed during the time of patients’ recruitment). Group allocation was forwarded to the training staff but was unknown to the study assistant performing the assessment at baseline and follow-up.

### 2.5. Measures

Short Physical Performance Battery (SPPB) was defined as the primary outcome and was assessed at baseline and max. 2 days after the last training intervention (before hospital discharge). SPPB assesses lower extremity physical performance status comprising the three subtests balance, walking speed, and chair rise. The time for performing the tasks in the SPPB is stopped in seconds. A score is then assigned to the respective duration with a maximum of 4 points in each subtest [18]. A change of 1 point has been shown to be of clinical relevance (e.g., predictive of future hospitalization, health improvement, and mortality [19].

The following secondary outcomes were also measured at baseline and after the intervention: The Barthel Index (BI) was determined for activities of daily life [20]. The Timed Up and Go (TUG) test was used to clinically assess patients’ mobility and fall risk [21]. The short Falls Efficacy Scale (short FES-I) was used to measure fear of falling [22]. The Parker Mobility Score (PMS) was used for the assessment of global mobility at baseline, at discharge, and during future telephone follow-up [23]. The treadmill treatment reports included total records, days, and treatment duration (hh:mm), walking speed (km/h), and number of perturbations (intervention group).

The baseline assessment was completed by the geriatric assessment routinely performed once at hospital admission. This includes patients’ sex, date of birth, weight and height. The age corresponds to the day of hospital admission. All outcomes were measured by assessors who were blinded concerning the patient’s group allocation. Falls were defined as an event that resulted in a person coming to rest inadvertently on the ground/floor or other lower level. Falls were reported as the number of falls during the previous 12 months. Hand grip strength, assessed with a hand dynamometer, was defined as the maximum value from three attempts. Cognitive function was assessed with the German Montreal Cognitive Assessment (MoCA) uncorrected for education [24]. The results of the MoCA were extrapolated according to the number of questions answered if the assessment could not be fully recorded for non-cognitive reasons (i.e., visual impairment). The Depression in Old Age Scale (DIA-S) was used for depressive symptoms [25], and the FRAIL Scale was used to identify persons at risk of frailty [26]. The German version of the SARC-F questionnaire was utilized to identify persons at risk of sarcopenia [27,28], and the Mini Nutritional Assessment—Short Form (MNA-SF) was used to assess the risk of malnutrition [29].

Due to prolonged recruitment during the COVID-19 pandemic, the follow-up period was concluded by the end of 2023, and data analysis is still ongoing at the time of manuscript submission.

### 2.6. Perturbation Treadmill

The treadmill (Balance TutorTM BT100, MediTouch, Tnuvot, Israel, https://meditouch.co.il/balancetutor/btsenior/, accessed on 23 September 2024) is a device to train reactive balance with individually adjustable perturbation movements in all four horizontal directions. It allows perturbations in 4 directions with 30 ascending degrees of strength. It allows patients to experience and train unexpected stumbling and slipping without the risk of falling. Patients are secured in a safety harness during training sessions, preventing a fall on the ground in case of an unsuccessful balance recovery. 

### 2.7. Interventions

The RCT was designed to compare perturbation-based balance training (PBT) on a treadmill with simple walking on an identical treadmill. All patients received the intervention on the treadmill in addition to usual care (“geriatric early rehabilitation” with medical treatment combined with a comprehensive geriatric assessment on admission, and physiotherapy and occupational therapy of a minimum of one hour per weekday over a period of 2 to 3 weeks, depending on the clinical circumstances). The intervention in both groups lasted at least 60 min in total with 4 or more sessions on the treadmill, aiming for 15 min per session, within 2 weeks of hospital stay. These 15 min sessions could be subdivided according to the individual performance level of the patient, e.g., into 3 × 5 min intervals. Each training session for the intervention group consisted of an individualized amount of unannounced and random unilateral treadmill belt accelerations, decelerations, and platform perturbations (shifts to the right or left side) while walking. Due to the specifics of the geriatric (multimorbid) study group, a standardized training program was not realistic and had to be individually adapted to the participants’ capacity. Thus, physiotherapists closely monitored the training. According to the patients’ individual capability, acceptance, and training progress, therapists adjusted walking speed; duration of the training; and (for the intervention group) frequency, directions, and strength of perturbations. Training started with strength level 3 and then, depending on the patient’s ability to regain balance after a perturbation, progressively increased the strength over the training sessions.

### 2.8. Changes from Planned Study

The first contact with the treadmill was walking without perturbations before randomization (and not after as initially planned) in order to get used to the system for about 5 min. In general, walking speed was individually set as a speed that was perceived as the usual walking speed by each participant.

### 2.9. Sample Size

The sample size was calculated based on data from Ramírez-Vélez [30]. The hypothesis for the current trial is that the intervention will improve the SPPB score by 1.0 points compared to the control group. A change of 1 point has been shown to be of clinical relevance (e.g., predictive of future hospitalization, health improvement, and mortality) [19]. The baseline value for SPPB was assumed to be 9.0 ± 2.0. The alpha level was set to 5% and the power to 80%. The sample size was calculated based on a *t*-test. Per group, *n* = 63 participants were needed to show a significant effect between treatment groups. Considering a dropout rate of 10%, *n* = 69 participants per group were actually needed (total sample of 138).

### 2.10. Statistical Methods

The statistical analysis was completed using SPSS statistical software (IBM SPSS, Version 29.0). Categorical variables are demonstrated as *n* (%). Means and standard deviations (SDs) were used for continuous data stratified by the study group. Primary analysis compared differences in the mean SPPB score (delta) between post-training and pre-training (baseline). A *t*-test was used to determine if there was a significant difference between groups in the SPPB score change between post-training and pre-training (baseline). 

Secondary analyses with the SPPB, TUG, PMA, BI, and fear of falling (short FES-I) were conducted with multiple linear regression analyses with change (delta post-training and pre-training) in each assessment as the outcome variable. Possible confounder variables were selected a priori as the most important known causal and conditional risk factors or variables significantly different between groups (study group, age, sex, and baseline value of assessments). Corresponding regression coefficients (b and their 95% CI) were calculated. The primary population under investigation included all patients randomized with at least one time-point following intention-to-treat (ITT) principles with missing values of the primary and secondary outcomes replaced by imputation procedures. Statistical significance was accepted with *p* < 0.05, and all CIs were computed at the 95% level. All patients who received one unit on the treadmill after randomization were included in the ITT analysis. As per protocol, analysis was performed considering adherence to at least 60 min of training.

## 3. Results

### Patients

**Figure 1:**
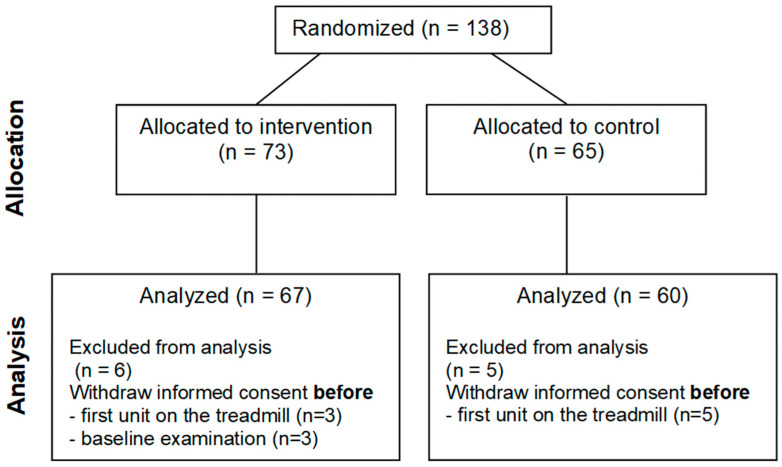
Trial profile.

Between October 2019 and December 2022, 138 patients were randomized (Figure 1).

Due to the COVID-19 pandemic, recruitment took significantly longer than initially planned. Two patients randomized to the control group mistakenly received perturbations (288 and 840 perturbations, respectively) and were left in the control group, in line with the ITT approach.

The baseline characteristics of the study participants are shown in Table 1. The study population consisted of 127 patients with a mean age of 81 (±6 standard deviation (sd)) years (range 70–94) with 81 (64%) women. The sex distribution was unbalanced, with a higher proportion of women but equal in both groups. All patients were multimorbid, and the main treatment diagnoses were 41 (32%) multifactorial immobility and gait disturbance; 28 (22%) falls (of which 12 (9%) with fractures); 9 (7%) vertigo of different origin; 8 (6%) lumbar spinal stenosis; 7 (6%) polyneuropathy; and 34 (27%) various diseases such as dementia, cerebral infarction, hyponatremia, Parkinson’s disease, polymyalgia, COVID-19, and osteoarthritis.

The mean number of falls in the past 365 days was 1.8 (±1.9, range 1–12). The mean score was 8 in the SPPB (±3, range 1–12), 70 in the BI (±13, range 40–100), 17.5 s in the TUG test (±6.9, range 7–40), and 7 in the PMS (±2, range 2–9). About one-third (29%) of all patients reported high concerns about falling in short FES-I, and 72% were classified as “frail” (FRAIL Scale). More than half of the patients demonstrated cognitive impairment (mean MoCA 21 ± 5 pts.). The average training involved 12 (±5) sessions over 6 (±2) days (meaning 2 sessions with a short break within 1 day) for 01:30 hh:mm (±00:42) as the total treatment duration. The total perturbation count was 609 (±286, range 7–1508) per patient, representing 1 perturbation every 10 s on average. There were no significant between-group differences in demographics and baseline characteristics of the patients. There were no adverse events due to the intervention.

Patients in the intervention group improved by a mean difference of 1.4 ± 2.1 in the SPPB score compared to 0.5 ± 1.7 in the control group with a difference of 0.9 points between the groups (delta). The comparison of the differences between the post-training and pre-training (baseline) of the primary outcome measure (SPPB) between the groups revealed a significant difference (*p* < 0.001) (Table 2). 

To elucidate the influence of the possible confounding variables on the primary outcome, the effect of the intervention was adjusted for other variables. The multiple linear regression analyses with differences in post–pre-SPPB as the dependent variable showed a significant association with the group variable (regression coefficient b = 0.63, 95% CI 0.02–1.23, *p* = 0.04) and the baseline SPPB but not with age; sex; or cognitive function, expressed as the MoCA score.

Therefore, the SPPB score of the participants in the intervention group improved significantly more than in the control group. Non-significant group differences concerning SPPB subtest gain could be detected in all three subtests, with 0.2 points more gain in the balance subtest, 0.2 points more gain in gait speed, and 0.4 points more gain in the chair rise subtest in the intervention group compared to the control group.

Multiple linear regression analyses with all secondary outcomes revealed no further significant associations. Sensitivity analyses with the per-protocol population showed no relevant differences compared to the ITT analysis.

## 4. Discussion

To the best of our knowledge, this is the first study analyzing the potential benefit of perturbation-based treadmill training in frail and prefrail geriatric hospitalized patients. The study provides evidence that this type of training is possible in this particular group, which has already been shown by a preliminary analysis of this study [31]. We now present data that demonstrate that this vulnerable group with a high fall risk experiences significant benefit from this training in terms of physical performance and lower extremity function, as measured by the SPPB. After a mean of 89 min treadmill training on six occasions during a mean hospital stay of 17 days, the SPBB score increased in both groups (0.5 pts. in the control group and 1.4 pts. in the intervention group) with a significant difference of 0.9 pts. (*p* < 0.001) in favor of the intervention group. A change of 1 point has been demonstrated to be of clinical relevance, so it can be assumed that patients benefit from the intervention.

However, secondary outcome measures such as fear of falling and the Barthel Index did not change significantly between the groups. In addition, differences in the reported fall rate during the follow-up at 3, 6, and 12 months have not yet been analyzed due to the ongoing data analysis.

The fact that the secondary outcomes of the study, such as fear of falling, remained unchanged is not unusual and is similar to the study by Gerards and colleagues [32]. Fear of falling is not an objective measure of performance but a subjective measure of fall-related concern. As such, it will probably take longer than two weeks to regain confidence in respective activities. In addition, a tailored approach should be used to achieve a higher level of perceived effectiveness and a reduction in fear of falling [33].

Up to now, perturbation-based balance training on a treadmill has been studied mainly in community-dwelling older adults [14,34] or in distinct disease categories such as Parkinson’s disease [35]. In the most recent study on community-dwelling older adults by Norgaard and colleagues [14], participants had a mean age of 72 years and were excluded if they had orthopedic surgery within the past 12 months, osteoporosis-related fractures, any progressive neurological disease, or severe cognitive impairment [34]. All these conditions were intentionally not excluded from our study to ensure the participation of subjects with the highest risk of falls and to be able to recruit and train a broader group of participants during a hospital stay. That is why our study population had, for instance, a higher mean age of 81 years, with 97% being prefrail or frail, and 86% with cognitive impairment (MoCA < 26 pts.). In the Norgaard study, the mean SPBB score was 12 points, whereas our study participants presented a mean of 8.6 points at baseline, all of which indicate a multimorbid and frail older hospital population in the current study.

According to the regression analysis, subjects with a lower SPBB score at baseline had a significantly more pronounced increase in the SPBB score because of the intervention. This may partly reflect the ceiling effect of the SPBB on the one hand, while on the other, it also demonstrates the fact that even subjects with poor functional performance gain substantial benefits from the perturbation-based treadmill training. Different from the study by Norgaard and colleagues, subjects with the entire range of SPBB scores from 1 to 12 were included, with 36% of subjects with an SPBB score below 8 points.

Interestingly, according to the regression analysis, cognitive function did not modify the treatment effect, i.e., subjects with lower cognitive function demonstrated the same improvement as those with normal or nearly normal cognitive function. This is of particular importance as many geriatric patients suffer from cognitive dysfunction of various etiologies, and it remains unclear if these subjects experience the same benefit from interventions, because they are mostly excluded from such studies. In this study, we included all patients who were able to follow instructions and give written informed consent. However, the point range of the MoCA score in the current study population was 5–29 pts., with 32 (26%) subjects showing a MoCA score below 18 pts., representing more than just mild cognitive impairment. From our point of view, it is of great importance to include subjects with cognitive impairment in fall prevention trials, because they make up more than half of the geriatric patient population, and particularly because they comprise a population with a markedly high fall risk. Cognitive impairment increases the risk of falls by 30% and increases the risk of fall-related injuries, including hip fractures and head injuries, by 100% [36,37].

With regard to the possible interventions in older persons with cognitive impairment, reactive balance training on a perturbation treadmill could be particularly attractive as the training does not necessarily involve planned actions and voluntary physical activity, except walking. Perturbation-based balance training on a treadmill purely relies on reactive actions due to the perturbations and is considered to be a highly effective form of training currently available [12].

However, an increase of 0.9 points in the SPPB score compared to the control group with 90 min of training within 2 weeks of hospital stay appears to be indicative of effective training in terms of gain per time invested. Other studies also consider this form of training to be highly effective, especially considering the relatively low training volume [7,8]. Nevertheless, the effect on fall prevention is yet to be proven in our population.

In comparison to other studies, the participants of this study were challenged by a very high number of perturbations, i.e., one perturbation every 10 s on average. On the other hand, the intensity of perturbations was comparatively moderate, as a compensatory extra step was not regularly observed, and this was not required in our individualized training protocol. To conclude, the manner in which physiotherapists performed the individualized training in our study could be described as high-frequency but low-intensity perturbation training. However, the optimal dosage and intensity of perturbations are not yet clear [6,38] and may not be unique for all patients in such a heterogenic group. Besides the tailored and individualized training intensity, Koschate et al. [39] emphasized that a unified method is needed to measure the success of the training. Nevertheless, adapting the intensity of perturbations to the individual limits of balance control, i.e., to challenge each participant progressively until compensatory steps are performed, would probably be an effective approach for most participants. That is why we currently recommend such a training strategy for future studies in geriatric patients.

### Limitations

This trial has several limitations. First, due to the nature of the intervention, participants and physiotherapists could not be blinded to group randomization, whereas assessors were blinded. However, due to the patients’ communication during discharge assessment and the ongoing follow-up interviews, the blinding of the assessors cannot fully be assured. Second, due to the heterogeneous study cohort and to ensure individualized training, we were not able to establish a highly standardized training protocol but defined minimum training requirements. Third, due to practical reasons, we did not measure reactive balance before and after the intervention in this first pilot trial involving geriatric patients. Fourth, due to the typically fluctuating cognitive function associated with underlying acute diseases [40], we were not able to reliably categorize patients with cognitive impairment into common cognitive disease categories. Therefore, we limited ourselves to reporting cognitive function on admission.

## 5. Conclusions

A total of 89 min of perturbation-based reactive balance training on a perturbation treadmill during hospital stay significantly increased physical performance in frail and prefrail hospitalized geriatric patients. However, its effectiveness on fall prevention still has to be proven.

## Figures and Tables

**Table 1:** Characteristics of the study participants (*n* = 127) at baseline and treatment report of intervention.

	All	Intervention Group (*n* = 67)	Control Group (*n* = 60)			
	*n* (%) or Mean ± sd	*n* (%) or Mean ± sd	*n* (%) or Mean ± sd	*p*	OR	95% CI
Age in years	81 ± 6	80 ± 5	81 ± 6	0.31	-	-
Female	81 (64)	44 (65)	37 (62)		0.89	0.41–1.90
Height (m) *	1.64 ± 0.1	1.63 ± 0.1	1.65 ± 0.1	0.19	-	-
Weight (kg) *	73 ± 16	72 ± 16	75 ± 16	0.30	-	-
Body mass index (kg/m^2^) *	27.3 ± 5.3	27.1 ± 5.1	27.5 ± 5.6	0.66	-	-
Duration of hospital stay (days)	17 ± 3	17 ± 3	17 ± 3	0.15	-	-
Falls previous 12 months	1.8 ± 1.9	1.9 ± 1.0	1.8 ± 2.2	0.71	-	-
Barthel Index (median) (pts.)	70 ± 13	69 ± 13	71 ± 12	0.31	-	-
Timed Up and Go (s)	17.5 ± 6.9	17 ± 7	17.7 ± 7.2	0.76	-	-
Hand grip strength max value (kg) *	20 ± 9	19 ± 9	20 ± 9	0.57	-	-
Montreal Cognitive Assessment (pts.) *	21 ± 5	20 ± 4	21 ± 5	0.89	-	-
Parker Mobility Score * (pts.)	7 ± 2	7 ± 2	7 ± 2	0.77	-	-
Short Physical Performance Battery (pts.) *	8.3 ± 2.6	8.1 ± 2.6	8.6 ± 2.7	0.38	-	-
Fear of falling (short FES-I) (pts.)	11.5 ± 4.7	11.7 ± 4.9	11.2 ± 4.5	0.55	-	-
low concern (7–8 pts.)	47 (37)	21 (31)	26 (43)			
moderate concern (9–13 pts.)	43 (34)	25 (38)	18 (30)			
high concern (14–28 pts.)	37 (29)	21 (31)	16 (27)			
Depression in Old Age Scale (DIA-S) (pts.)	2.8 ± 2.4	2.7 ± 2.5	2.9 ± 2.3	0.73	-	-
no depression (0–2 pts.)	70 (55)	40 (60)	30 (50)		-	-
depression suspected (3 pts.)	14 (11)	5 (7)	9 (15)		-	-
probable depression (4–10 pts.)	43 (34)	22 (33)	21 (35)		-	-
FRAIL Scale	3.5 ± 1.6	3.4 ± 1.7	3.6 ± 1.5	0.45	-	-
robust (<1 pt.)	4 (3)	4 (6)	0		-	-
prefrail (1–2 pts.)	32 (25)	16 (24)	16 (27)		-	-
frail (>2 pts.)	91 (72)	47 (70)	44 (73)		-	-
Mini Nutritional Assessment—Short Form (MNA-SF) *	9.4 ± 2.3	9.3 ± 2.2	10.2 ± 3.5	0.63	-	-
normal nutritional status (12–14 pts.)	25 (20)	12 (18)	13 (22)		-	-
at risk of malnutrition (8–11 pts.)	74 (58)	39 (58)	35 (58)		-	-
malnourished (0–7 pts.)	27 (21)	15 (22)	12 (20)		-	-
Sarcopenia (SARC-F)	4.2 ± 2.0	4.1 ± 2.1	4.3 ± 1.9	0.69	-	-
low risk (0–3 pts.)	54 (43)	33 (49)	21 (35)		-	-
high risk (4–10 pts.)	73 (58)	34 (51)	39 (65)		-	-
Treatment Report						
Total days	6 ± 2	7 ± 2	6 ± 2	0.07	-	-
Total treatment duration in hh:mm	01:29 ± 0:41	1:34 ± 0:43	1:23 ± 0:39	0.14	-	-
Minimal walking speed (km/h)	1.0 ± 0.4	1.0 ± 0.4	1.0 ± 0.4	0.35	-	-
Maximal walking speed (km/h)	1.5 ± 0.7	1.4 ± 0.6	1.6 ± 0.8	0.55	-	-
Total perturbations ^+^	-	609 ± 286	564 ± 390 (*n* = 2) ^#^		-	-

* 1–2 missing values; ^+^ only intervention group (*n* = 67); ^#^ incorrectly assigned after randomization. no: numbers, sd: standard deviation, pts.: points.

**Table 2:** Differences in post- and pre-training characteristics of the study participants (*n* = 127).

	Difference Post–Pre-Training		Difference in Groups		
	Intervention Group (*n* = 67)	Control Group (*n* = 60)	*t*-Test		
	Mean ± sd	Mean ± sd	Mean	95% CI	*p*
Primary Outcome					
Short Physical Performance Battery (pts.)	1.4 ± 2.1	0.5 ± 1.7	−0.9	−1.6, −0.2	<0.001
Secondary Outcomes					
Timed Up and Go (s)	−4.6 ± 5.2	−4.4 ± 5.3	0.2	−1.6, 2.1	0.8
Parker Mobility Score (pts.)	0.1 ± 0.9	0.2 ± 0.9	0.1	−0.3, 0.4	0.7
Barthel Index (pts.)	16.7 ± 14.3	13.0 ± 9.1	−3.9	−8.2, 0.4	0.1
Fear of falling (short FES-I) (pts.)	−1.0 ± 2.8	−1.0 ± 2.8	0	−1.0, 1.0	1.0

s: seconds, sd: standard deviation, pts.: points.

## Data Availability

The data can be obtained from the corresponding author upon request.
